# Donor Kidney Recovery Methods and the Incidence of Lymphatic Complications in Kidney Transplant Recipients

**Published:** 2010-02-01

**Authors:** R. F. Saidi, J. A. Wertheim, P. Kennealey, D. S. C. Ko, N. Elias, H. Yeh, M. Hertl, T. Kawai

**Affiliations:** Department of Surgery, Transplantation Unit, Massachusetts General Hospital, Harvard Medical School, Boston, MA, USA

**Keywords:** kidney transplantation, surgery, complications, lymphatic leak

## Abstract

Background: Lymphatic leak and lymphocele are well-known complications after kidney transplantation.

Objective: To determine the incidence of lymphatic complications in recipients of living donor kidneys.

Methods: Among 642 kidney transplants performed between 1999 and 2007, the incidence of lymphatic complications was retrospectively analyzed in recipients of living donor kidneys procured by laparoscopic nephrectomy (LP, n=218) or by open nephrectomy (OP, n=127) and deceased donor kidneys (DD, n=297). A Jackson-Pratt drain was placed in the retroperitoneal space in all recipients and was maintained until the output became less than 30 mL/day.

Results: Although the incidence of symptomatic lymphocele, which required therapeutic intervention, was comparable in all groups, the duration of mean±SD drain placement was significantly longer in the LP group—8.6±2.7 days compared to 5.6±1.2 days in the OP group and 5.4±0.7 days in the DD group (p<0.001). Higher output of lymphatic drainage in recipients of LP kidneys could lead to a higher incidence of lymphocele if wound drainage is not provided.

Conclusion: More meticulous back table preparation may be required in LP kidneys to decrease lymphatic complications after kidney transplantation. These observations also support the suggestion that the major source of persistent lymphatic drainage following renal transplantation is severed lymphatics of the allograft rather than those of the recipient’s iliac space.

## INTRODUCTION

Lymphatic leak and lymphocele are well-known complications after kidney transplantation, occurring among 0.6%–22% of the recipients [1-4]. The recent increase of living donation in kidney transplantation may be attributed to the nation-wide expansion of LP. We introduced laparoscopic donor nephrectomy in 1999 and currently, essentially all donor nephrectomies are performed laparoscopically. However, we observed apparently prolonged period of lymphatic drainage in kidney transplant recipients who received the laparoscopically procured kidney. Therefore, in the current study, we systematically reviewed and compared the duration of lymphatic drainage and the incidence of lymphatic complications among the kidney transplant recipients who received living donor kidneys, procured by either LP or OP, or those from DD. As demonstrated by lymphangiography, two sources of lymphatic leak have been proposed: injured lymphatics in the recipient’s iliac space and injured lymphatics of the kidney graft [3, 4]. Ligation of all major lymphatic channels at the time of skeletonizing recipient’s iliac vessels has been recommended to minimize the risk of these complications [3, 4]. In this study, we focused on the other source of the lymphatic leak—the kidney allograft—and evaluated the lymphatic complications in recipients who received kidney grafts procured by three different donor recovery methods—laparoscopic nephrectomy (LP), open nephrectomy (OP) and deceased donor (DD) kidneys. 

## MATERIAL AND METHODS

We retrospectively analyzed 642 patients who underwent kidney transplantation at our institution from January 1999 to March 2007. Among these recipients, 218 received living donor kidneys procured by LP, 127 received those procured by OP and 297 recipients received those from DD. Previously described standard kidney recovery methods were employed [8, 9].

During DD recovery and open live donor nephrectomy, lymphatics of renal hilum were meticulously ligated using silk suture. On the other hand, ultrasonic shear was used for dissection during the laparoscopic donor nephrectomy with less back table preparation in the LP group. A Jackson-Pratt drain was placed in the retroperitoneal space in all transplant recipients. The recipients were typically discharged from the hospital on day 4–5, regardless of the output of the drain. The drain was maintained until the output became less than 30 mL/day with no evidence of fluid collection by ultrasound examination.

One-way ANOVA was used to compare the mean in the three study groups. A p value <0.05 was considered statistically significant.

## RESULTS

**Figure 1:**
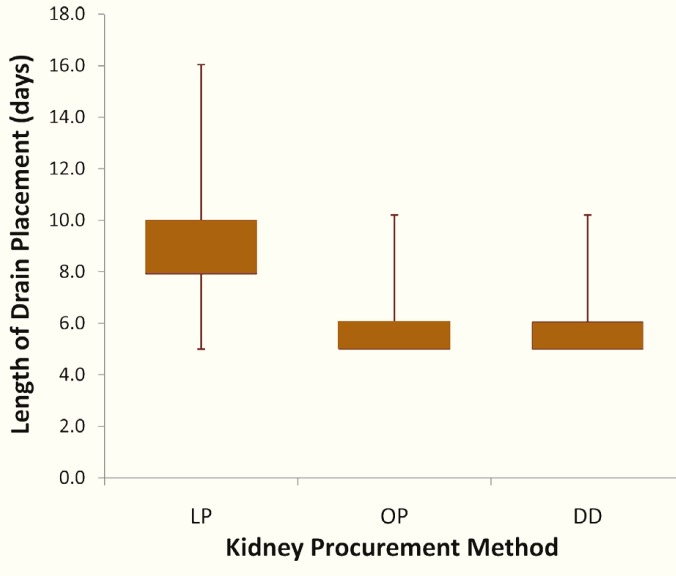
Whisker-box plot of length of drain placement

The mean±SD duration of drain placement was significantly longer in the LP group—8.6±2.7 days, compared to 5.6±1.2 days in the OP group and 5.4±0.7 days in the DD group (Fig. 1). Although the longer drain placement did not require longer hospital stay (Table 1), more recipients (41.7%) in the LP group were discharged home with the drain in place, compared to the OP group (19.6%) and DD group (20.2%). Following removal of the initial drain, the incidence of symptomatic lymphocele which required therapeutic interventions was comparable in all groups (Table 1).

**Table 1:** Lymphatic complications after kidney transplantation stratified by organ recovery method

Parameter	Living donor Laparoscopic (n=218)	Living Donor Open (n=127)	Deceased Donor (n=297)
Mean±SD hospital stay (days)	5.1±0.07	5.2±1.4	8.7±5.4*
Discharge with drain (%)	91 (41.7%)*	25 (19.6%)	60 (20.2%)
Mean±SD duration of drain Placement (days)	8.6±2.7*	5.6±1.2	5.4±0.7
Symptomatic lymphocele (%)	7 (3.2%)	4 (3.1%)	9 (3.0%)

* p<0.001

## DISCUSSION

Lymphatic leak and lymphocele formation after kidney transplantation are usually attributed to injured lymphatics in the recipient caused by extensive dissection around the iliac vessels or disrupted lymphatics in the donor kidney [1, 2]. Heparin, corticosteroids, and early patient mobilization have also been suggested as contributory factors [2-4]. Although prolonged lymphatic leakage usually resolves without intervention, percutaneous drainage followed by either sclerotherapy or internalization of the lymphocele into the peritoneal cavity are required to treat symptomatic lymphocele [10, 11]. 

Laparoscopic live donor nephrectomy has increased in popularity since its inception in 1995 [5]. Many studies have demonstrated the advantages of LP over OP, which include decreased post-operative pain, shorter hospital stay and recuperation time, and improved cosmetic result [5-7]. The LP group showed a significantly longer duration of drain placement (8.6 days) compared to 5.6 days in the OP and 5.4 days in the DD groups. Although longer drain placement did not require longer hospital stay, more patients in the LP group were discharged from the hospital with the drain in place because of persistently high output from the drain. In the DD group, the length of drain placement was comparable to the OP group, but their length of hospital stay appeared longest due to delayed function of the transplanted kidney. One explanation for these findings would be the different techniques of hilar dissection used during the kidney recovery. During LP, ultrasonic shears were mainly used to dissect the hilum of the donor kidney. In contrast, in our standard OP, meticulous ligation of the hilar tissues is typically performed. In DD kidneys, similar meticulous ligation of lymphatics is performed during the back table preparation. In contrast, there have been no differences in the recipient procedure or perioperative management among these recipients. Thus, the donor recovery method is the only apparent surgical factor that might explain the differences observed in lymphatic drainage among these groups. Our practice is to routinely drain the retroperitoneal space during the early post-operative period. This approach makes the prolonged drainage in the recipients who received the laparoscopically procured kidney immediately evident. Although prolonged lymphatic drainage resolved in the most patients without intervention, it would presumably increase the risk of symptomatic lymphocele if a retroperitoneal drain was not placed. Based on these observations, our protocol for LP has been modified by now performing more meticulous ligation of lymphatics in the kidney hilum on the back table. 

In conclusion, the duration of lymphatic leak was longer in recipients who received laparoscopically procured kidney graft. These observations support the suggestion that lymphatic leakage following renal transplantation originates primarily from the kidney graft rather than from severed lymphatics in the recipient iliac fossa. More meticulous ligation of perihilar tissue of the kidney graft, especially in the laparoscopically procured kidneys, may decrease the lymphatic complications after kidney transplantation. 
